# A rare complication of intracardiac double knotting of temporary pacemaker lead during bedside insertion: a case report

**DOI:** 10.1093/ehjcr/ytae623

**Published:** 2024-11-28

**Authors:** Aditi Dattagupta, Shweta Agrawal, Srilakshmi Adhyapak, Harshith Kramadhari, Abhilash Konda

**Affiliations:** Department of Cardiology, St. John’s National academy of Health Sciences, University-Rajiv Gandhi University of Health Sciences, Bengaluru 560034, Karnataka, India; Department of Cardiology, St. John’s National academy of Health Sciences, University-Rajiv Gandhi University of Health Sciences, Bengaluru 560034, Karnataka, India; Department of Cardiology, St. John’s National academy of Health Sciences, University-Rajiv Gandhi University of Health Sciences, Bengaluru 560034, Karnataka, India; Division of interventional Radiology, Kasturba Medical College, Manipal University-Manipal academy of Higher Education, Manipal 576104, Karnataka, India; Formerly Assistant Professor, Department of Radiology, St. John’s National academy of Health Sciences, Bengaluru 560034, Karnataka, India; Department of Cardiology, St. John’s National academy of Health Sciences, University-Rajiv Gandhi University of Health Sciences, Bengaluru 560034, Karnataka, India

**Keywords:** Temporary pacemaker lead, Knots, Snare, Case report

## Abstract

**Background:**

Temporary pacemaker lead implantation is a common low-risk procedure, but can occasionally get complicated by infections, arrhythmias, thromboembolic events, and perforation of the vessel or the heart. However, intracardiac knotting of the temporary pacemaker lead has been rarely reported. This could lead to vascular or valvular injury, pneumothorax, symptomatic loss of pacing or haemodynamic compromise, and difficult lead removal.

**Case summary:**

We are reporting a case of twice twice-knotted temporary pacemaker lead, which to our knowledge has not been reported before. The two knots in the transjugularly inserted temporary pacemaker lead, via a 6F venous sheath made it difficult to retrieve it.

**Discussion:**

We decided to snare the knotted TPI into the inferior vena cava, and then retrieve it via a large-size femoral sheath, thus avoiding the need for a venotomy or any surgical intervention.

**Learning points**

- Avoid over manipulation of a temporary pacemaker lead, knotting is a very likely possibility due to over rotation especially in bedside insertions.
- A relatively easy procedure like transvenous temporary pacemaker implantation can turn into a nightmare if the lead gets knotted, twisted, risks damaging the tricuspid valve apparatus.
- Snaring the lead into the more roomy inferior vena cava and then out through a large-size venous sheath is a feasible option rather than vein cut down or vascular surgery intervention.

## Introduction

Temporary pacemaker lead implantation is a common low-risk procedure, but can occasionally get complicated by infections, arrhythmias, thromboembolic events, and perforation of the vessel or the heart. However, intracardiac knotting of temporary pacemaker lead has been rarely reported. This could lead to vascular or valvular injury, pneumothorax, symptomatic loss of pacing or hemodynamic compromise, and difficult lead removal.^1^ We are reporting a case of twice twice-knotted temporary pacemaker lead, which to our knowledge has not been reported before.

## Summary figure

**Figure:**
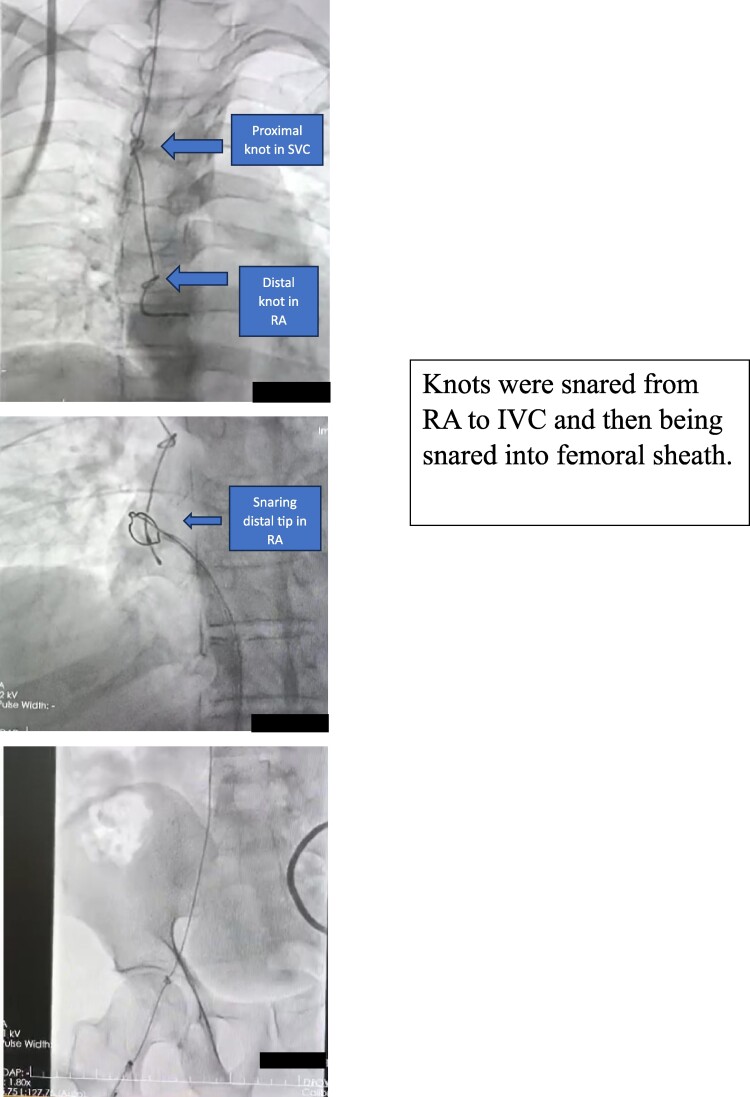


## Case report

**Figure 1:**
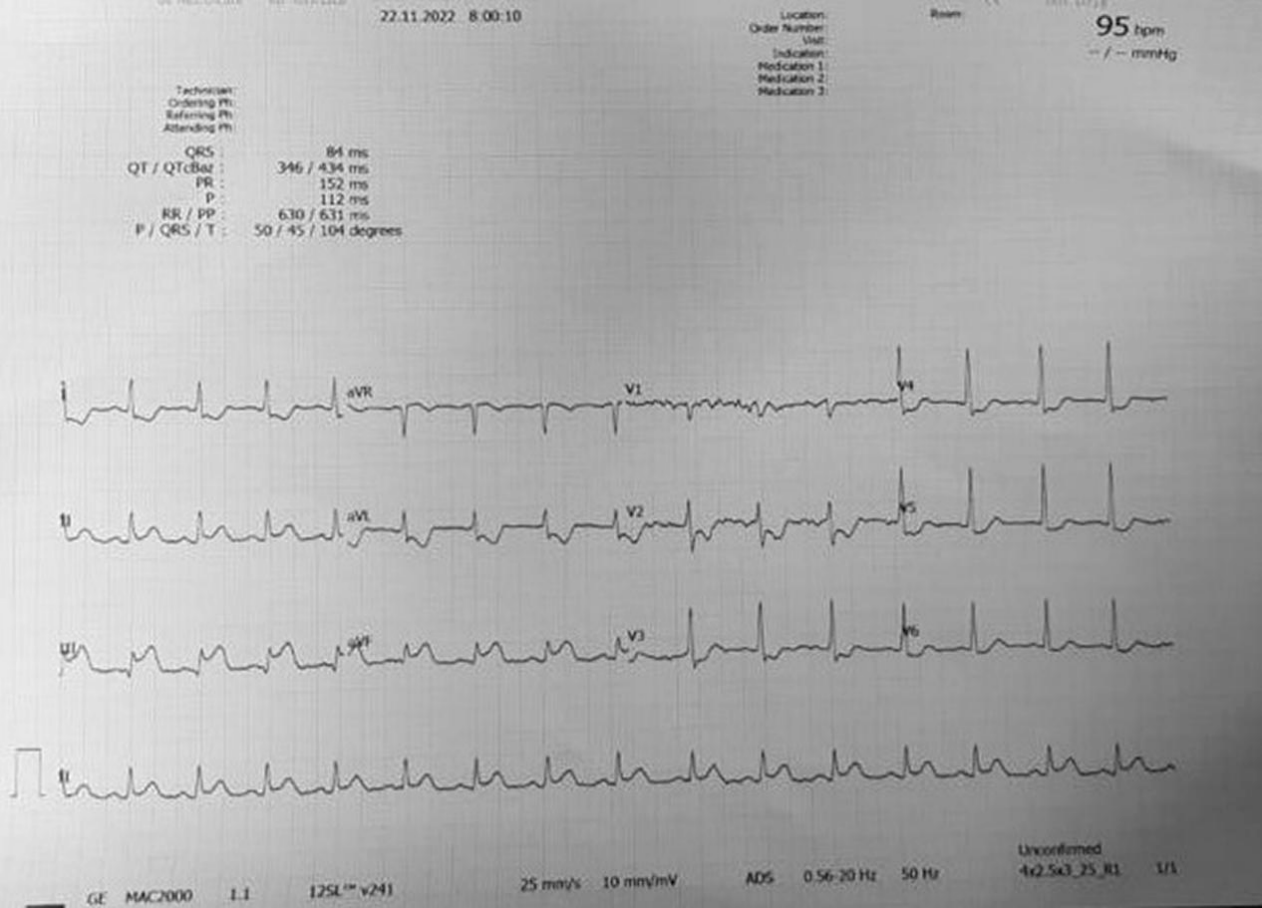
Admission ECG showing ST segment elevation in inferior leads.

A 47-year-old gentle man came to Emergency room with a history of chest pain for 2 days. His ECG showed ST elevation with T inversion in II, III, aVF leads and hence was diagnosed as Evolved Inferior Wall ST-elevation MI (*Figure 1*). 2D-Echocardiography revealed hypokinesia of inferior wall with mild LV dysfunction, LVEF of 47%. He was taken for early PCI. Coronary angiography revealed RCA having early bifurcation with total occlusion of PL branch and PTCA with stenting was done to PL Branch with a DES. Procedure went uneventful and patient was hemodynamically stable.

**Figure 2:**
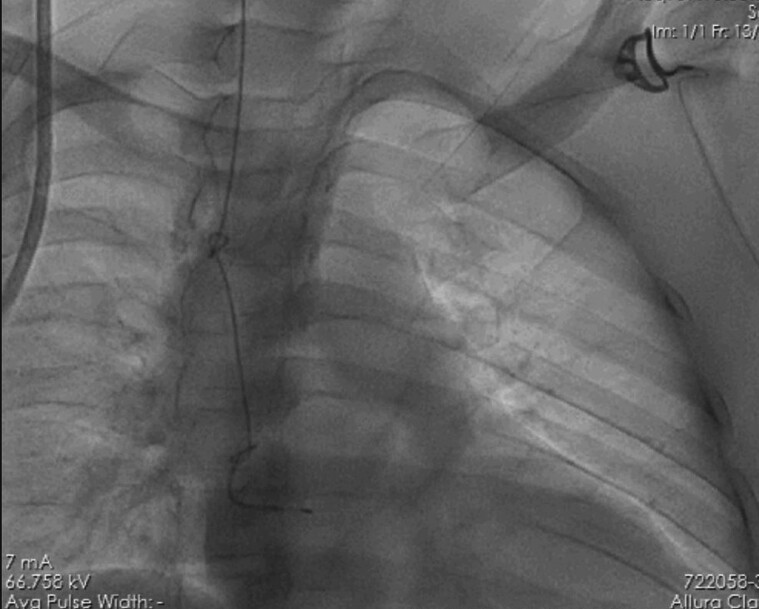
Two knots in the temporary pacemaker lead are visible on fluoroscopy, one in the superior vena cava (SVC) and the second in the right atrium (RA).

Next day morning, patient developed intermittent complete heart block and AV block with narrow QRS complex and heart rate of 50 beats/minute. Varying degrees of AV block, including transient complete heart block is commonly seen in acute Inferior wall myocardial infarction patients. Although patient was asymptomatic, check angiography was planned and back-up temporary pacemaker insertion was attempted bed-side through right Internal Jugular vein under Echocardiography guidance, by the on-duty cardiology resident. After repeated attempts, pacing was not achieved and there was inability to pull TPI lead out for reattempt, hence, patient was taken to cath-lab for fluoroscopy, followed by check angiogram. On fluoroscopy, temporary pacemaker lead was found to have two knots, one in Right Atrium and one in Superior Vena Cava (*Figure 2*).

**Figure 3:**
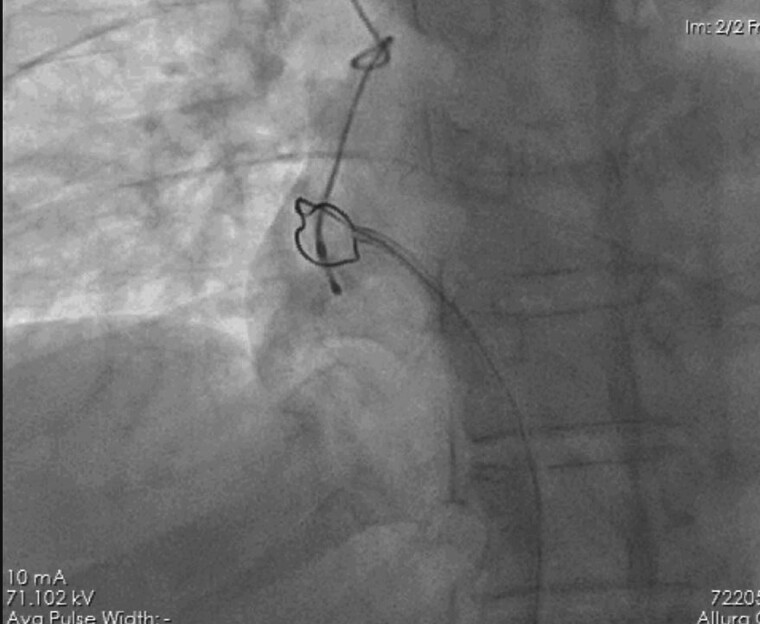
Distal tip of temporary pacemaker lead is being caught by a snare under fluoroscopic guidance.

Attempt was made to undo the knots through torquing the TPI lead which was unsuccessful. Thereafter, a 14 Fr long sheath was introduced through Right Femoral Vein. A 25 mm ONE Snare (Merit Medical) endovascular snare was introduced through sheath and tip of knotted lead was securely grasped and pulled down into the IVC (*Figure 3*).

**Figure 4:**
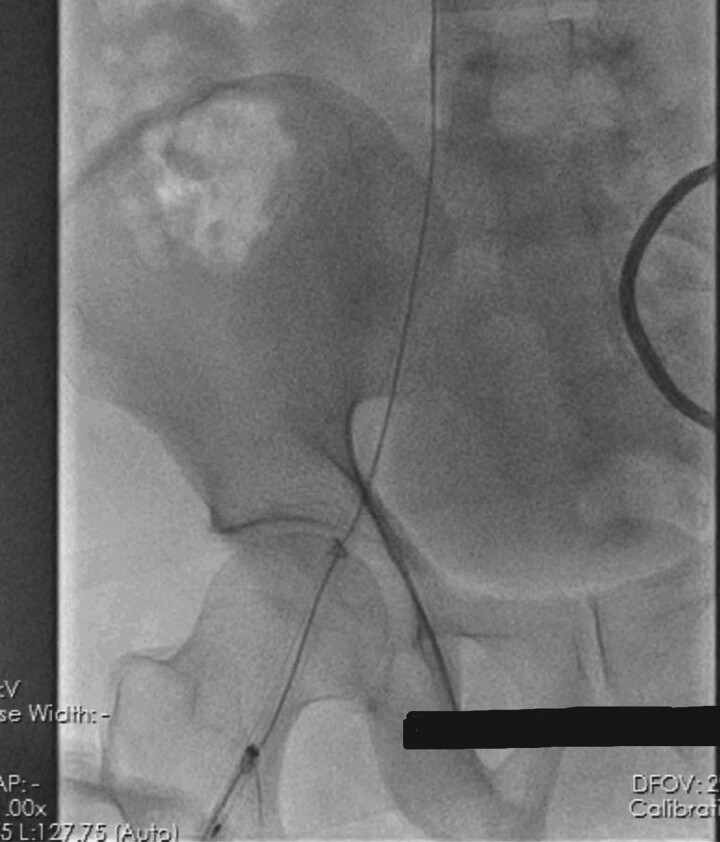
Temporary pacemaker lead with two knots is being snared into the femoral sheath.

**Figure 5:**
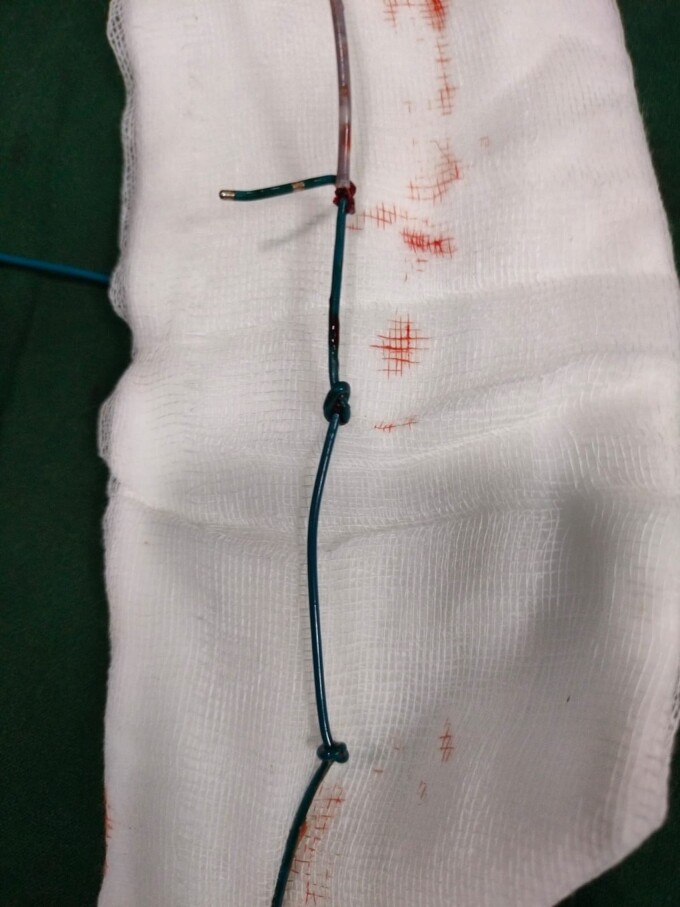
Two knots seen, after retrieval of the lead.

As snare was not stable to pull the lead upto the sheath, long sheath was inserted and again lead tip was snared. Knots were tightened by tensing from proximal end (outside the right internal jugular vein) and snared end and was pulled into the tip of long sheath. Then proximal external portion of the jugular pacing lead was cut and thoroughly cleansed with sterile normal saline. Entire assembly was gently pulled back and removed into the long sheath, making the knot increasingly smaller and facilitating complete extraction (*Figures 4* and *5*).

The procedure was completed without any need for venotomy.

Check angiography revealed patent stent and no distal embolization. Within 24 h, complete heart block reverted to sinus rhythm and patient was later discharged in a stable condition.

## Discussion

Intracardiac knotting of temporary pacing lead is not a frequently encountered complication. Knotting can occur with various other devices like cardiac catheters (which have a central lumen), guidewires etc.^2^ The temporary pacemaker lead consists of a non-braided polymer which is radiopaque, and lacks a central lumen, which adds to its stiffness making it less likely to kink and form a knot.

Intracardiac knot formation can occur due to supple temporary pacemaker lead, as well as the lack of technical experience of operators.^3^ Flexibility of the lead is beneficial while inserting the temporary lead, but it can be hazardous since its redundancy may lead to formation of loops during manipulation and can entangle tricuspid valve apparatus including its chordae tendineae and can cause puckering of cusps.^4^

One should avoid overzealous torquing or manipulation especially during bedside insertion of temporary pacemaker implantation. Echocardiography guidance and using balloon-tipped floating catheter TPI, help in correct placement, especially in bedside emergency situations where patient cannot be shifted to cath lab. However, balloon-tipped floating catheter is more expensive and is not available in all centres. Even with echocardiography guidance, being mindful of the insertion length of the TPI, would give an approximate idea if the lead tip is placed correctly or has lot of deflections. The bedside ECG monitor also gives clues as one may see premature ventricular complexes when one has entered the right ventricle.

First reported case of an intravascular catheter knot was by Johansson *et al.* in 1954.^5^ Various methods of unknotting can be used, for example, putting a long sheath through the same route and covering the knot with the sheath, use of snares, retrieval baskets, endomyocardial biopsy forceps, and angioplasty balloon inflation.^6^

Most knots can be untied percutaneously using simple manoeuvres. In case of catheters with central lumen, insertion of a guidewire would help to unravel the knot. Unlooping of knot, by passing a guidewire through the loop followed by balloon dilatation^7,8^ or hooking and pulling it with a 0.03500 J tipped guide wire^9^ or with a pigtail catheter^2^ is also described. Surgical removal might be required especially if knot is large in size or with many loops and not getting tightened by snare or develops intracardiac fixation or causing intracardiac damage.

As temporary pacing does not have any central lumen and two knots in our case made its retrieval more difficult than lumen catheters. One approach described is to tighten the knot as much as possible and remove it through vein of insertion, but this approach required venotomy.^2,10^ Another method is by holding the distal end of the knotted lead with a snare and pulling it back and forth by simultaneously holding its proximal end.^2,11^

So the options for retrieval were, one we try to tighten the knot as much as possible and then pull it out of the insertion sheath. However, this too would have required a separate access for snaring and since the insertion sheath was a 6F Internal jugular vein sheath (caliber not large enough to allow TPI lead knots into its lumen), forcefully pulling the knot into it would have had high chances of vessel damage and failure. Another option would have been Venotomy and retrieval. Since our patient had undergone coronary angioplasty with stenting less than 24 h ago and was on dual antiplatelets with heparin, we wanted to avoid venotomy. Also, venotomy would have been a more invasive procedure, requiring vascular surgeon on board, and additional time to make arrangements.

As there were two knots in our case, and in order to avoid venotomy, the strategy used in our case was to make the knot tight by pulling the distal end with snare through right femoral vein, and then bring it in IVC smoothly. Then, the knots were pulled into a longer sheath, withdrawing it enmass through femoral venous access site after cutting the lead’s proximal end.

In our case, temporary lead knotting could have occurred due to excessive manipulation without fluoroscopic guidance due to emergency situation by relatively inexperienced operator. Although echo cardiography guidance was being used, and did show the pacemaker lead in the RA, however clearly making out damage or knot on echocardiography image is not possible. Attempt was made once to undo the knots through torquing the TPI lead, which was unsuccessful, and because of two knots, untying was not tried further. Also tightening of knots and pulling out through vein of insertion was not attempted as two knots had to be navigated through vein (*Figure 5*). On initial fluoroscopy, one knot was seen in SVC and other in RA just over tricuspid valve. Immediately decision was taken to try and bring the knots into IVC to prevent the knot from entering into RV and causing any rent in RA due to excessive manipulation in order to untie knots. Hence Femoral venous sheath of 14 Fr was introduced and distal tip of temporary lead was snared into IVC, after which knots were tightened enough to be pulled out of 14 Fr sheath.

## Follow up

The patient’s subsequent stay in hospital was uneventful. He remained in normal sinus rhythm thereafter. He continues to follow up as outpatient last 2 years and has been doing well with no episodes of angina or syncope.

## Conclusion

Knotting of temporary pacing lead is infrequent, and getting two knots is even rarer. This can be prevented by approximating the length of lead required to be inserted to reach right ventricle, inserting lead under fluoroscopy guidance, and avoid forceful insertion against resistance. Such knotted leads can be successfully retrieved percutaneously by reducing the knot size by tightening it by pulling its distal end with snare, and then snaring it into a long sheath and withdrawing the whole assembly after cutting its proximal end. One can avoid surgical intervention and hence complications and additional financial burden to the patient.

## Supplementary Material

ytae623_Supplementary_Data

## Data Availability

The data underlying this article are available in the article and in its online Supplementary material.

## Lead author biography

I, Dr Aditi Dattagupta, graduated with my primary medical degree (MBBS) in 2005. Thereafter, I completed my 3 years of residency in Internal Medicine in 2009. I have completed my training (3 years of residency) in the super-speciality of Cardiology in 2017, from Apollo Hospital, Chennai, India. I am currently working as Assistant Professor in the Department of Cardiology in St. John’s National Academy of Health sciences, Bengaluru, India. My areas of interest are Acute coronary syndrome, coronary interventions, heart failure, dyslipidaemia, and Cardiovascular imaging.

## Supplementary material

**Consent**: The manuscript has been submitted after obtaining due consent from the patient as per COPE guidelines. The original consent has been retained by our institute, a scanned copy of the consent has been mailed to the Editor of the journal.
