# Supplementary figures and images for: A rare complication of intracardiac double knotting of temporary pacemaker lead during bedside insertion: a case report
Source: Eur Heart J Case Rep. 2024 Nov 28;8(12):ytae623. doi: 10.1093/ehjcr/ytae623 (PMC11652086; doi:10.1093/ehjcr/ytae623)

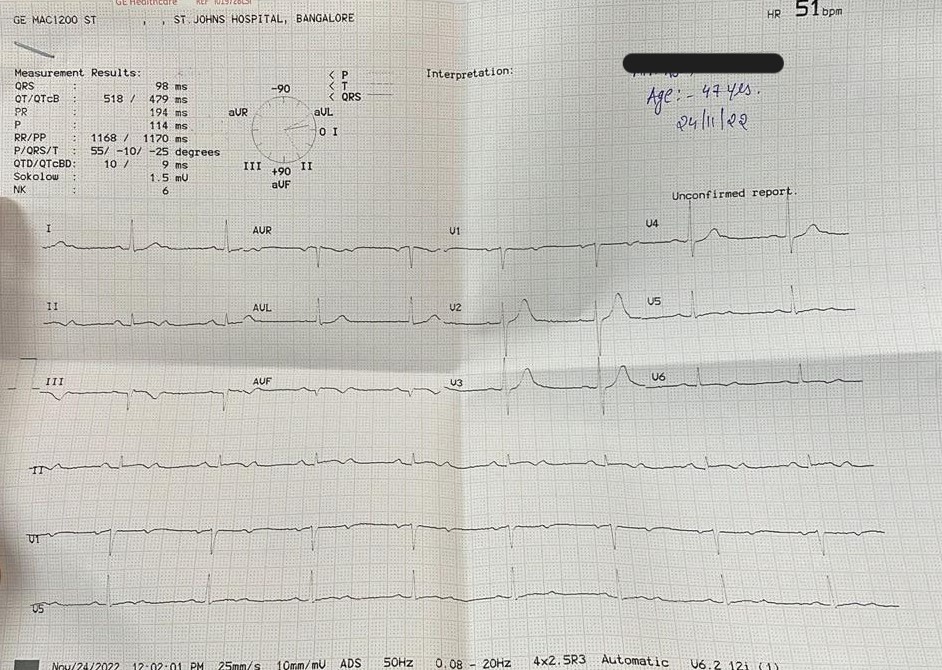

Supplement: ytae623_Supplementary_Data [file ytae623_supplementary_data.zip › Figure 2 - knotted TPI - AV block ECG.jpeg]
